# Effects of a Losartan-Antioxidant Hybrid (GGN1231) on Vascular and Cardiac Health in an Experimental Model of Chronic Renal Failure

**DOI:** 10.3390/nu15081820

**Published:** 2023-04-10

**Authors:** Laura Martínez-Arias, Sara Fernández-Villabrille, Cristina Alonso-Montes, Gonzalo García-Navazo, María P. Ruíz-Torres, Ramón Alajarín, Julio Alvarez-Builla, Elena Gutiérrez-Calabres, Juan José Vaquero-López, Natalia Carrillo-López, Diego Rodríguez-Puyol, Jorge B. Cannata-Andía, Sara Panizo, Manuel Naves-Díaz

**Affiliations:** 1 Bone and Mineral Research Unit, Instituto de Investigación Sanitaria del Principado de Asturias (ISPA), Hospital Universitario Central de Asturias (HUCA), Universidad de Oviedo, 33011 Oviedo, Spain; lauramartinezarias@gmail.com (L.M.-A.); sarafv0012@gmail.com (S.F.-V.); cristinaam.huca@gmail.com (C.A.-M.); jorge.cannata@gmail.com (J.B.C.-A.); sarapanizogarcia@gmail.com (S.P.);; 2 Redes de Investigación Cooperativa Orientadas a Resultados en Salud (RICORS), RICORS2040 (Kidney Disease), 28040 Madrid, Spain; mpiedad.ruiz@uah.es (M.P.R.-T.); elena.gutierrez@edu.uah.es (E.G.-C.); juanjose.vaquero@uah.es (J.J.V.-L.); 3 Departamento de Química Orgánica y Química Inorgánica, Instituto de Investigación Química “Andrés M. del Río” (IQAR), Universidad de Alcalá (IRYCIS), 28805 Alcalá de Henares, Spain; gonzalo.garcia@uah.es (G.G.-N.); alajarinramon@gmail.com (R.A.); julio.alvarez@uah.es (J.A.-B.); 4 Physiology Unit, Department of Systems Biology, Facultad de Medicina y Ciencias de la Salud, Universidad de Alcalá, Área 3-Fisiología y Fisiopatología Renal y Vascular del Instituto Ramón y Cajal de Investigación Sanitaria (IRYCIS), Instituto Reina Sofía de Investigación Nefrológica (IRSIN) de la Fundación Renal Iñigo Álvarez de Toledo (FRIAT), 28871 Alcalá de Henares, Spain; 5 Departamento de Medicina, Universidad de Alcalá, Servicio de Nefrología, Hospital Universitario Príncipe de Asturias, 28871 Alcalá de Henares, Spain

**Keywords:** cardiovascular, renal failure, antioxidant, losartan, inflammation

## Abstract

Drugs providing antihypertensive and protective cardiovascular actions are of clinical interest in controlling cardiovascular events and slowing the progression of kidney disease. We studied the effect of a hybrid compound, GGN1231 (derived from losartan in which a powerful antioxidant was attached), on the prevention of cardiovascular damage, cardiac hypertrophy, and fibrosis in a rat model of severe chronic renal failure (CRF). CRF by a 7/8 nephrectomy was carried out in male Wistar rats fed with a diet rich in phosphorous (0.9%) and normal calcium (0.6%) for a period of 12 weeks until sacrifice. In week 8, rats were randomized in five groups receiving different drugs including dihydrocaffeic acid as antioxidant (Aox), losartan (Los), dihydrocaffeic acid+losartan (Aox+Los) and GGN1231 as follows: Group 1 (CRF+vehicle group), Group 2 (CRF+Aox group), Group 3 (CRF+Los group), Group 4 (CRF+Aox+Los group), and Group 5 (CRF+GGN1231 group). Group 5, the CRF+GGN1231 group, displayed reduced proteinuria, aortic TNF-α, blood pressure, LV wall thickness, diameter of the cardiomyocytes, ATR1, cardiac TNF-α and fibrosis, cardiac collagen I, and TGF-β1 expression. A non-significant 20% reduction in the mortality was also observed. This study showed the possible advantages of GGN1231, which could help in the management of cardiovascular and inflammatory processes. Further research is needed to confirm and even expand the positive aspects of this compound.

## 1. Introduction

Angiotensin-converting enzyme inhibitors have been widely used in the treatment of hypertension. Some beneficial effects of antihypertensive molecules have been attributed, at least partially, to their antioxidant ability [1], due to the well-known mechanism of oxidative stress in developing vascular damage [2]. In fact, previous data from our group demonstrated that the use of several losartan-antioxidant hybrids was able to block the angiotensin II effect with increased antioxidant ability. In hypertensive rats, these hybrids were able to control hypertension and prevent hypertension-induced cardiovascular damage better or as much as losartan [3,4].

Hence, the search for drugs that can provide antihypertensive effect together with other protective cardiovascular actions is of clinical interest, particularly in patients with chronic kidney disease (CKD) [5,6] in which cardiac injury induces cardiac remodelling characterized for the increase in the size of cardiomyocytes, while fibroblasts increase collagen synthesis that leads to fibrosis. This process leads to apoptosis or necrosis of cardiomyocytes which are replaced by fibroblasts and extracellular collagen with the consequent progression of fibrosis [7,8]. Experimental induction of moderate and severe chronic renal failure (CRF) leads not only to the development of cardiac hypertrophy and fibrosis [9,10] but also to vascular damage that, in the last instance, might be conducive to the appearance of vascular calcification [11].

Thus, the aim of this work was to study the effect of the hybrid compound GGN1231 on the prevention of cardiovascular damage, cardiac hypertrophy and myocardial fibrosis using an experimental animal model of severe CRF. GGN1231 is derived from losartan, to which a powerful antioxidant, dihydrocaffeic acid, was attached.

## 2. Materials and Methods

### 2.1. Experimental Studies

**Figure 1:**
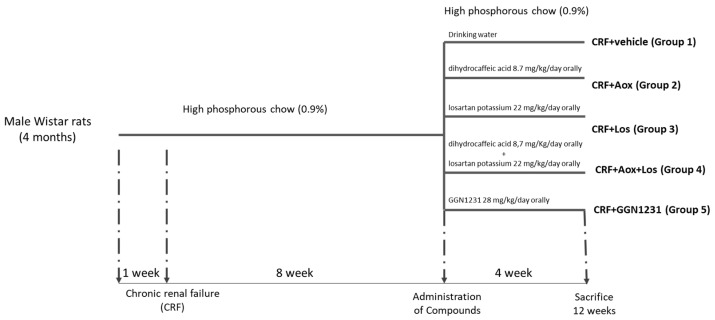
Experimental design of the rat model of chronic renal failure fed a high phosphorus diet. CRF (chronic renal failure); Aox (antioxidant); Los (losartan); Aox+Los (antioxidant plus losartan); GGN1231 (antioxidant plus losartan hybrid).

The design of the study is detailed in Figure 1. Male Wistar rats (350–400 g) were housed in wire cages in a controlled environment with a 12-h light/dark cycle and water and food ad libitum. The rats were fed with a standard diet rich in phosphorous (0.9%) and normal calcium (0.6%) (Panlab, Barcelona, Spain) for a period of one week to be familiar with the diet before the induction of CRF by 7/8 nephrectomy as previously detailed [12].

After the nephrectomy, rats were maintained for a period of 12 weeks with a high phosphorus diet to aggravate the progression and severity of CRF [13] (Figure 1). In week 8, rats were randomized into five groups receiving different drugs during the following four weeks. Group 1 (CRF+vehicle or Control group) received 1 mL of ethanol dissolved in drinking water. Group 2 (CRF+antioxidant (Aox), CRF+Aox group) received 8.7 mg/kg/day of dihydrocaffeic acid (Sigma-Aldrich, San Luis, CA, USA). Group 3 (CRF+losartan (Los), CRF+Los group) received 22 mg/kg/day of losartan potassium (AK Scientific, Union City, NJ, USA). Group 4 (CRF+antioxidant+losartan, CRF+Aox+Los group) received 8.7 mg/kg/day of dihydrocaffeic acid and 22 mg/kg/day of losartan potassium. Group 5 (CRF+GGN1231 group) received 28 mg/kg/day of GGN1231, using 8.7 mg/kg/day of dihydrocaffeic acid and 22 mg/kg/day of losartan potassium for manufacturing the compound. The dose of GGN1231 used was, according to previous study, those needed to reduce blood pressure [3]. All drugs were dissolved in 1 mL of ethanol and added to the drinking water.

**Figure 2:**
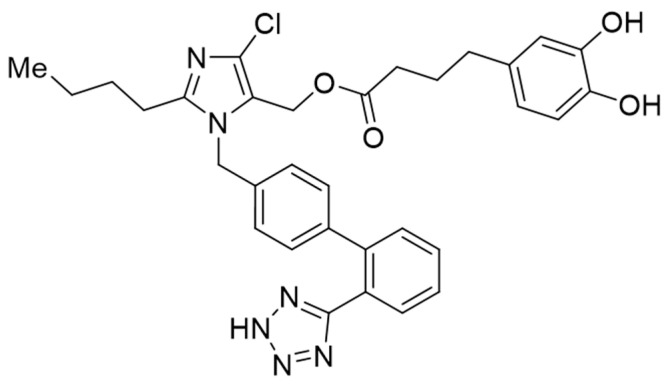
Structure of GGN1231 compound.

The GGN1231 was synthesized in the Organic and Inorganic Chemistry Department of Alcala University, Spain (Figure 2). It is a hybrid compound obtained by adding an antioxidant fragment, dihydrocaffeic acid, to the hydroxymethyl side chain of losartan [3,4].

Systolic (SBP) and diastolic (DBP) blood pressure were measured before the first administration of the drugs in weeks 8 and 12 before sacrifice, and the day before, the rats were introduced into metabolic cages for 24-h urine collection. Rats were weighed and euthanized by exsanguination using isofluorane anesthesia. Serum and urine samples were drawn for analysis, and the hearts were removed, washed twice with saline solution, blotted dry and weighed. The left ventricle (LV) was then divided into two pieces: one section was frozen in liquid nitrogen and stored at −80 °C for RNA, and the other section was fixed and embedded in paraffin for histological studies. Aortas were removed and washed twice with saline solution. One fragment was used for RNA extraction and to determine the calcium content.

The protocols were approved by the Research Ethics Committee of Oviedo University (PROAE 15/2015).

### 2.2. Analytical and Technical Procedures

#### 2.2.1. Biochemical Markers

Serum creatinine, calcium, phosphorus, and urinary creatinine, calcium, phosphorus, and protein were measured using a multichannel auto-analyzer (Hitachi 717; Boehringer Mannheim, Germany). Serum intact PTH (iPTH) was measured by IRMA (Rat PTH kit Immutopics, San Juan Capistrano, CA, USA), following the manufacturer’s protocols.

#### 2.2.2. Aortic Calcium Content

A frozen aortic fragment was homogenized in 0.6 N NaCl and stirred at 4 °C for 24 h. Upon centrifugation, calcium content was determined colorimetrically in the supernatant by the o-Cresolphtalein-Complexone method (Sigma-Aldrich, San Louis, CA, USA) [14]. The remaining aortic pellet was resuspended in lysis buffer (125 mM Tris, 2% SDS, pH 6.8) for protein extraction and quantification. Calcium content, normalized for total protein, was expressed as mg calcium/mg protein.

#### 2.2.3. Blood Pressure Measurement

Before the initiation of the administration of the drug (week 8) and before the sacrifice (week 12), SBP and DBP were measured using an automated, non-invasive tail-cuff method (LSI Letica, Barcelona, Spain). In order to minimize the procedure-induced stress, the animals were accustomed to the instrument for four consecutive days prior to the definitive measurements, which consisted of a set of a minimum of 10 repetitive measurements per rat.

#### 2.2.4. Cardiac Morphological and Histological Changes

Morphological and histological changes in the heart, left ventricular (LV) wall, septum thickness and cardiomyocyte diameter were measured in deparaffined 3 µm sections. They were stained with hematoxylin-eosin (Sigma-Aldrich, San Louis, CA, USA) and visualized using an optical microscope (model DMRXA2, Leica Microsystems, Wetzlar, Germany) coupled to a digital video camera (model Dc-100, Leica Microsystems, Wetzlar, Germany). Captured images were evaluated using an image analysis system (Image J). The mean cardiomyocyte diameter was determined by measurement of transnuclear widths of random, longitudinally oriented in 20 myocytes with magnification 40×. The LV wall and septum thickness were measured using pre-design software, which pooled and analyzed a set of at least 50 blinded radius measurements from the center of the LV to its outer edge.

The myocardial total collagen area was determined by using Masson’s trichrome, using a semiautomatic image analysis software (Leica QWIN standard version 2.3, Leica Microsystems, Wetzlar, Germany). The measurements were blinded, and the results were expressed as percentages of the total myocardial area. The collagen fiber/muscular tissue ratio was calculated.

#### 2.2.5. Immunohistochemistry

Transforming growth factor 1 (TGF-β1) localization was assessed by immunohistochemistry in 3 µm sections. Samples were deparaffinized, rehydrated and incubated at 98 °C, pH 6 (Dako EnVision Flex Retrieval Solution Low pH, Dako, Glostrup, Denmark) for 30 min and blocked with 3% of bovine serum albumin (BSA) in PBS for 1 h. After overnight incubation at 4 °C with primary antibody of transforming growth factor beta 1 (TGF-β1) (1:100, AB92486, Abcam, Cambridge, UK) in 3% BSA (Sigma-Aldrich, San Luis, CA, USA) in PBS, slices were washed and incubated with a biotinylated secondary antibody following the manufacturer’s instructions (Dako REAL EnVision, Glostrup, Denmark).

A negative control without a primary antibody was used to set the level of the lowest detectable staining intensity. Along with Masson’s trichrome staining, semiautomatic image analysis software (Leica QWIN standard version 2.3, Leica Microsystems, Wetzlar, Germany) was used. Briefly, the image of each heart was converted to grayscale; then, using the optical density function of the software, pixels that fell within a designed threshold were counted, obtaining a mean value of grey color density. TGF-β1 staining was expressed as the average optical density.

#### 2.2.6. RNA Extraction, cDNA Synthesis, and Quantitative RT-PCR

Aorta and LV fragments were homogenized in an Ultraturrax (OmniHT). Total RNA was extracted by the TRIzol method (Sigma, Saint Louis, CA, USA). Total RNA concentration and purity were quantified by UV-Vis spectrophotometry (NanoDrop Technologies, Wilmington, NC, USA), measuring its absorbance at 260 and 280 nm. Reverse transcription was performed with a High-Capacity cDNA Reverse Transcription Kit (Applied Biosystems, Foster City, CA, USA) following the manufacturer’s instructions.

Gene expression was measured in the aorta and heart by qRT PCR using an ABI Prism 7000 Sequence Detection System (Applied Biosystems, Waltham, MA, USA). TaqMan Real-time PCR amplification was performed with gene-specific primer (Gene Expression Assays from Applied Biosystems, Waltham, MA, USA) for α-actin, RUNX Family Transcription Factor 2 (Runx2) and tumor necrosis factor-alpha (TNF-α) in the aorta. TaqMan Real-time PCR amplification was performed for angiotensin receptors 1 and 2 (ATR1 and ATR2), Mas receptor, collagen I, TGF-β1, and TNF-α in the heart. Rat glyceraldehyde-3-phosphate-dehydrogenase (GAPDH) was used in both tissues as a housekeeping gene. The relative quantitative evaluation of the target gene was performed by comparing threshold cycles using the ΔΔCt method [15].

### 2.3. Statistical Analysis

Results were expressed as a median and interquartile range in the tables and mean in the graphics. For all variables, the Mann–Whitney U test was applied to validate the existence of significant differences from group to group. Significant differences were considered when *p* < 0.05. The statistical program used was R 4.2.0.

## 3. Results

### 3.1. Effect of Dihydrocaffeic Acid, Losartan, and Dihydrocaffeic Acid plus Losartan and GGN1231 on Weight, Biochemical, and Urinary Markers, and Vascular and Inflammation Parameters

Table 1 shows there were no differences in weight, serum calcium, phosphorus, iPTH, creatinine, creatinine clearance (Cr Cl), urinary calcium, and phosphate among groups. In the CRF+Los and the CRF+GGN1231 groups, proteinuria was significantly lower than in the CRF+vehicle group (control group) and CRF+Aox group (Table 1). No changes at this level were observed in the CRF+Aox+Los group.

No significant differences in aortic calcium content were found among the groups, though a non-significant reduction in aortic calcium content (4-fold) compared to the CRF+vehicle group was observed in the CRF+GGN1231 group (Table 2). The aortic gene expression of Runx2 and α-actin showed no significant differences, but a significant difference in mRNA TNF-α was observed between the GGN1231 group and the other four groups (Table 2).

### 3.2. Effect of Dihydrocaffeic Acid, Losartan, Dihydrocaffeic Acid plus Losartan and GGN1231 on Blood Pressure, Left Ventricular Hypertrophy, Cardiac Inflammation, Cardiac Fibrosis, and Survival

**Figure 3:**
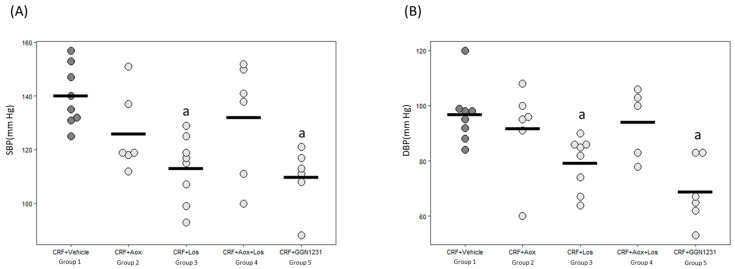
(**A**) Systolic (SBP) and (**B**) diastolic (DBP) blood pressure. CRF (chronic renal failure); Aox (antioxidant); Los (losartan); Aox+Los (antioxidant plus losartan); GGN1231 (antioxidant plus losartan hybrid). Data represent mean values per group in horizontal lines in the bee swarm graphic. ^a^ *p* < 0.05 versus CRF+vehicle.

**Figure 4:**
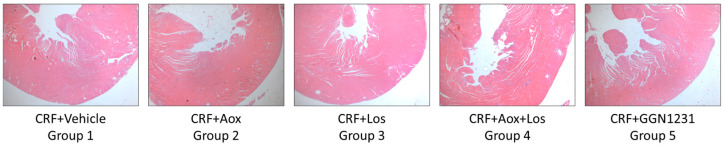
Representative images of hematoxylin-eosin staining for the hearts used to analyze the parameters of cardiac hypertrophy are described in Table 3. CRF (chronic renal failure); Aox (antioxidant); Los (losartan); Aox+Los (antioxidant plus losartan); GGN1231 (antioxidant plus losartan hybrid).

The SBP and DBP were significantly lower in the CRF+Los and CRF+GGN1231 groups compared with the control group (Figure 3). Figure 4 shows representative images of hematoxylin-eosin staining for the hearts used to analyze the parameters of cardiac hypertrophy described in Table 3. A trend to decrease the heart weight/body weight ratio was observed in the CRF+Los and CRF+GGN1231 groups. In the latter, a significant reduction of the LV wall, but not of the septum thickness, was observed (Table 3).

The cardiomyocyte diameter was significantly lower in the four groups receiving the different compounds compared to the control group (CRF+vehicle, *p* = 0.017, *p* = 0.0029, *p* < 0.001, and *p* = 0.002, respectively) (Table 3).

In the four groups treated with the different compounds, the ATR1 increase was prevented (*p* = 0.008, *p* = 0.024, *p* = 0.006, and *p* = 0.002, respectively). No effect was observed neither in the ATR2 nor in the MAS receptor (Table 4). The four compounds used were able to decrease cardiac TNF-α (*p* < 0.005) (Table 4).

**Figure 5:**
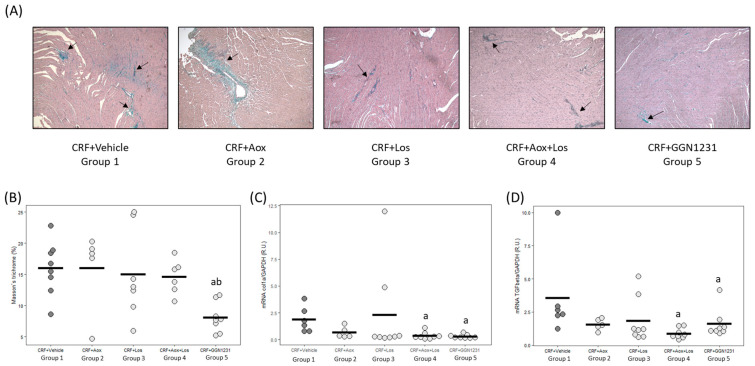
Cardiac fibrosis: Masson’s trichromic staining and Collagen I and TGF-β1 gene expression evaluated by qPCR. (**A**) Representative photos of Masson’s trichrome staining (10×). The arrows point to areas with fibrosis. (**B**) Masson’s trichrome staining quantification in all rats. (**C**) Collagen I (Col I) mRNA. (**D**) TGF-β1 mRNA. CRF (chronic renal failure); Aox (antioxidant); Los (losartan); Aox+Los (antioxidant plus losartan); GGN1231 (antioxidant plus losartan hybrid). Data represent mean values per group in horizontal lines in the bee swarm graphic. ^a^ *p* < 0.05 versus CRF+vehicle; ^b^ *p* < 0.05 versus CRF+Aox.

**Figure 6:**
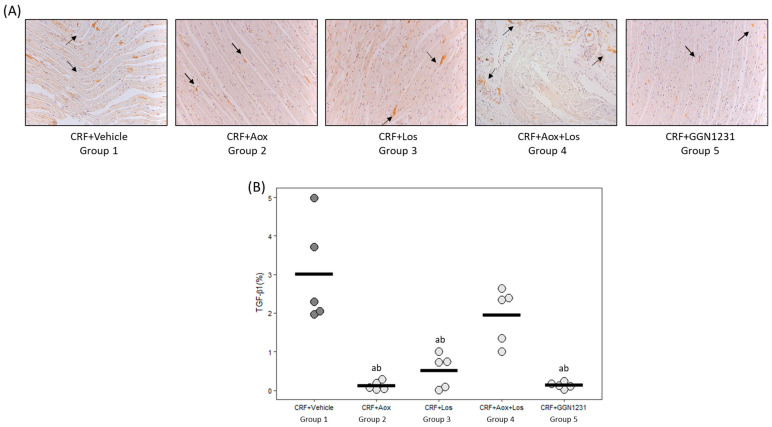
Protein expression of TGF-β1 by immunohistochemistry. (**A**) Representative photo of TGF-β1 immunohistochemistry (20×). (**B**) Arrows point to TGF-β1 protein expression Quantification of TGF-β1 immunohistochemistry in all groups. CRF (chronic renal failure); Aox (antioxidant); Los (losartan); Aox+Los (antioxidant plus losartan); GGN1231 (antioxidant plus losartan hybrid). Data represent mean values per group in horizontal lines in the bee swarm graphic. ^a^ *p* < 0.05 versus CRF+vehicle; ^b^ *p* < 0.05 versus CRF+Aox+Los.

The CRF+GGN1231 group showed a significant reduction in cardiac fibrosis (Figure 5A,B), and gene expression of collagen I and TGF-β1 (Figure 5C,D), with the latter also reduced in the CRF+Aox+Los group. In the CRF+vehicle group, the protein expression of TGF-β1 by immunohistochemistry was 22-fold higher than in the CRF+GGN1231 group (Figure 6A,B).

The survival of the four groups receiving compounds was slightly but not significantly higher than the control group (CRF+vehicle); 10% in the CRF+Aox, CRF+Los and CRF+Aox+Los groups and a 20% in the CRF+GGN1231 group.

## 4. Discussion

This pilot study showed that the new compound, GGN1231, was able to reduce proteinuria, blood pressure, LV wall thickness, the diameter of the cardiomyocytes, ATR1, cardiac TNF-α, and cardiac fibrosis showing a decrease in collagen I gene expression and TGF-β1 gene and protein expression. None of the drugs was able to reduce heart weight, septal thickness, ATR2 and MAS receptor. At the vascular level, no differences were found either in aortic calcium content or in the molecular transition from vascular smooth muscle cells to bone cells. A reduction in the vascular inflammatory markers was only observed in the CRF+GGN1231 group. The four groups receiving compounds showed a non-significant decrease in mortality ranging from 10% (CRF+Aox, CRF+Los, and CRF+Aox+Los groups) to 20% (CRF+GGN1231 group).

A well-known action of losartan is its ability to reduce proteinuria [16,17], and this effect was also observed in the present study when losartan was given separately or combined with an antioxidant as a single compound (GGN1231). The antiproteinuric effect observed could be partly explained due to the control of hypertension. In fact, previous studies in hypertensive Wistar rats showed a reduction in blood pressure with the GGN1231 compound, like the response to losartan [4]. Nevertheless, the antiproteinuric effect of losartan could also be independent of hypertension and may be related to the changes in glomerular hemodynamics [18] associated with the reduction of the glomerular protein leakage [19], and the size of unselective pores in the glomerular basement membrane [20].

Losartan is a selective antagonist of ATR1 used in the treatment of hypertension, which could also act on angiotensin II (Ang II) through a competitive mechanism [21] upregulating the expression of the converting enzyme (ACE) or inhibiting Ang II production in situ [22]. Losartan also upregulates myocardial expression of ACE2, catalyzing Ang II to form angiotensin 1–7 [23], which then binds Mas receptors carrying a protective function [8,24]. As expected, in our study, the CRF+Los and CRF+GGN1231 groups showed a reduced SBP, DBP, and ATR1 gene expression, which was more marked in the CRF+GGN1231 group. The administration of dihydrocaffeic acid (CRF+Aox group), a powerful and natural antioxidant derived from caffeic acid, also showed a significant reduction in the ATR1 gene expression. In fact, previous studies have shown that caffeic acid modulates the renin-angiotensin-aldosterone endocrine axis [25] and lowers SBP and plasma ACE activity in hypertensive rats [26], though this effect has not been observed in rats with CRF [27]. In contrast, no effect was observed on ATR2 and Mas receptor gene expression with any of the other four compounds.

Several clinical and experimental studies have shown that losartan reduces LVH and fibrosis [28,29]. The maintained increase in Ang II increases TGF-β1 levels in cardiomyocytes and fibroblasts [30,31], having autocrine and paracrine effects by stimulating TGF-β1 receptors. The latter leads, among other effects, to increasing inflammatory signals, hypertrophy of cardiomyocytes, and proliferation and synthesis of extracellular matrix components, such as collagen and fibronectin [32,33]. Caffeic acid has also shown anti-inflammatory [34] and cardioprotective properties decreasing cardiomyocyte damage and apoptosis [35]. In our study, losartan, dihydrocaffeic acid and GGN1231 reduced the cardiomyocytes’ size, but only the latter decreased wall thickness. The combination of losartan and dihydrocaffeic acid in one hybrid compound, GGN1231, showed a lower degree of cardiac fibrosis (5A, 5B3) than losartan or dihydrocaffeic acid administered separately or even when both were given together and at the same time (CRF+Aox+Los group). In fact, in the latter group, the protective effect of losartan to control blood pressure and proteinuria was lost. Several drugs are known to be inhibitors of the cytochrome P450 hepatic enzymes, which may influence the response to drug combinations or drug dosages. There are no reports about the possible interaction between losartan and dihydrocaffeic acid or any caffeic acid [36], though some studies have shown interactions of some herbs with losartan [37,38]. Thus, it can be speculated that antioxidants may modulate the activity of cytochrome P450 and, thus, decrease some of the properties of the losartan.

The main reason to produce and test the hybrid compound of losartan and dihydrocaffeic acid, GGN1231, was to consider the possibility of driving losartan and the antioxidant (molecularly bound to losartan) to the specific receptors of losartan to evaluate if it would be possible to have, at the tissue level, the protective effects of the antioxidant and losartan. The results obtained with this compound showed some beneficial effects beyond those offered by the administration of each of the same compounds administered separately or even when they are administered at the same time. The benefits of GGN1231 were mainly observed in vascular inflammation, cardiac fibrosis and hypertrophy. In addition, the four compounds showed a non-significant reduction in the mortality rate, which was more manifest with GGN1231 (20% decrease vs. 10% decrease in the other groups). In agreement with these results, previous studies described that losartan reduced 10% of the mortality in different models [39,40].

The study has limitations. The small number of animals studied may have prevented finding differences in clinically relevant outcomes such as vascular calcification and the possibility of obtaining stronger results. In addition, four weeks of study could have been insufficient to obtain significant changes among groups. Despite the mentioned limitations, the study showed interesting possible advantages of the new hybrid compound GGN1231, which could help in the management of cardiovascular and inflammatory processes. Further research is needed to confirm and even expand the positive aspects of the new compound observed in this pilot study.

## 5. Conclusions

Although all compounds improved some cardiovascular parameters, only the CRF+GGN1231 group was able to reduce aortic TNF-α, LV wall thickness and cardiac fibrosis leading to a non-significant 20% reduction in mortality. This study showed the possible advantages of GGN1231, which could help in the management of cardiovascular and inflammatory processes. Further research is needed to confirm and even expand the positive aspects of this compound.

## Figures and Tables

**Table 1:** Weight and biomarkers of kidney disfunction.

	CRF+Vehicle Group 1 (*n* = 8)	CRF+Aox Group 2 (*n* = 8)	CRF+Los Group 3 (*n* = 8)	CRF+Aox+Los Group 4 (*n* = 8)	CRF+GGN1231 Group 5 (*n* = 8)
Weight (g)	388 [375–407]	387 [371–401]	373 [352–375]	388 [382–396]	374 [365–398]
Creatinine (mg/dL)	2.1 [1.7–2.9]	1.9 [1.6–2.7]	1.7 [1.3–3.7]	2.2 [1.6–4.0]	2.1 [1.4–2.6]
Calcium (mg/dL)	10.1 [9.3–10.4]	9.3 [8.4–9.3]	9.5 [9.2–10.2]	9.6 [8.1–9.7]	8.8 [8.1–9.1]
Phosphorus (mg/dL)	12.2 [9.6–14.4]	12.3 [11.1–14.1]	10.1 [6.9–19.7]	13.5 [9.7–22.2]	14.0 [11.7–15.5]
PTH (pg/mL)	6745 [4975–7168]	6706 [6538–7331]	6169 [4395–6480]	6389 [5057–7072]	6608 [6403–6961]
Creatinine clearance (mL/min)	0.5 [0.4–0.8]	0.5 [0.4–0.6]	0.7 [0.5–0.9]	0.5 [0.2–0.6]	0.4 [0.3–0.6]
Creatinine clearance (mL/min/kg)	1.4 [0.9–1.7]	1.2 [0.8–1.8]	1.8 [0.6–2.3]	1.4 [0.9–1.7]	1.1 [0.9–1.8]
Proteinuria (mg/24 h)	74 [41–823]	92 [67–101]	19 [12–30] ^a,b,c^	66 [37–92]	22 [19–29] ^a,b,c^
Urinary calcium (mg/dL)	5.3 [3.5–5.9]	4.9 [3.2–7.3]	5.3 [3.2–5.9]	4.1 [2.9–6.6]	4.1 [3.4–6.2]
Urinary phosphorus (mg/dL)	109 [104–198]	109 [106–180]	174 [117–192]	157 [140–198]	138 [85–176]

CRF (chronic renal failure); Aox (antioxidant); Los (losartan); Aox+Los (antioxidant plus losartan); GGN1231 (antioxidant plus losartan hybrid); PTH (parathyroid hormone). Data represent median and interquartile range. ^a^ *p* < 0.05 versus CRF+vehicle; ^b^ *p* < 0.05 versus CRF+Aox; ^c^ *p* < 0.05 versus CRF+Aox+Los.

**Table 2:** Vascular and inflammation parameters.

	CRF+Vehicle Group 1 (*n* = 8)	CRF+Aox Group 2 (*n* = 8)	CRF+Los Group 3 (*n* = 8)	CRF+Aox+Los Group 4 (*n* = 8)	CRF+GGN1231 Group 5 (*n* = 8)
Calcium (µg de Ca/mg protein)	25.2 [6.1–73]	8.73 [6.7–8.9]	11.1 [6.2–44]	11.2 [7.7–15.3]	6.8 [4.0–8.7]
mRNA RUNX2/GAPDH (R.U.)	2.8 [1.6–7.0]	6.5 [3.0–9.2]	2.7 [0.7–9.3]	3.0 [2.2–5.2]	3.2 [1.2–4.4]
mRNA α-ACTIN/GAPDH (R.U.)	0.2 [0.1–0.3]	0.5 [0.3–1.5]	0.1 [0.1–0.2] ^b,c^	0.3 [0.2–0.5]	0.1 [0.1–0.6]
mRNA TNF-α/GAPDH (R.U.)	6.5 [1.6–8.8]	4.6 [4.6–8.0]	3.3 [1.66.45]	5.9 [2.5–7.3]	0.6 [0.5–2.1] ^a,b,c,d^

CRF (chronic renal failure); Aox (antioxidant); Los (losartan); Aox+Los (antioxidant plus losartan); GGN1231 (antioxidant plus losartan hybrid). Data represent the median and interquartile range. ^a^ *p* < 0.05 versus CRF+vehicle; ^b^ *p* < 0.05 versus CRF+Aox; ^c^ *p* < 0.05 versus CRF+Aox+Los; ^d^ *p* < 0.05 versus CRF+Los.

**Table 3:** Parameters of cardiac hypertrophy.

	CRF+Vehicle Group 1 (*n* = 8)	CRF+Aox Group 2 (*n* = 8)	CRF+Los Group 3 (*n* = 8)	CRF+Aox+Los Group 4 (*n* = 8)	CRF+GGN1231 Group 5 (*n* = 8)
Heart/body weight (mg/g)	4.8 [3.8–5.2]	4.9 [4.2–7.0]	3.4 [2.7–4.8]	4.2 [3.7–4.7]	3.6 [3.3–4.0]
LV Wall (μm)	2539 [2443–2786]	2364 [2199–2406]	2477 [2285–2665]	2396 [2330–2455]	2107 [2027–2215] ^a^
Septum (μm)	2125 [2041–2207]	2025 [1987–2601]	1861 [1767–2179]	1995 [1906–2141]	2012 [1892–2183]
Cardiomyocytes diameter (μm)	11.9 [11.0–12.4]	9.3 [8.9–10.6] ^a^	10.1 [8.7–10.5] ^a^	8.9 [8.7–9.6] ^a^	9.0 [8.6–9.4] ^a^
Heart/body weight (mg/g)	4.8 [3.8–5.2]	4.9 [4.2–7.0]	3.4 [2.7–4.8]	4.2 [3.7–4.7]	3.6 [3.3–4.0]

LV Wall (left ventricular wall); CRF (chronic renal failure); Aox (antioxidant); Los (losartan); Aox+Los (antioxidant plus losartan); GGN1231 (antioxidant plus losartan hybrid). Data represent the median and interquartile range. ^a^ *p* < 0.05 versus CRF+vehicle.

**Table 4:** Molecular markers of the renin-angiotensin system by quantitative PCR.

	CRF+Vehicle Group 1 (*n* = 8)	CRF+Aox Group 2 (*n* = 8)	CRF+Los Group 3 (*n* = 8)	CRF+Aox+Los Group 4 (*n* = 8)	CRF+GGN1231 Group 5 (*n* = 8)
mRNA ATR1/GAPDH (R.U.)	2.5 [2.1–2.7]	0.7 [0.4–0.8] ^a^	0.5 [0.4–0.8] ^a^	0.5 [0.4–0.6] ^a^	0.3 [0.3–0.5] ^a^
mRNA ATR2/GAPDH (R.U.)	5.6 [2.6–15.2]	12.7 [6.0–20.8]	4.7 [4.1–39.6]	14.8 [5.5–19.9]	8.7 [4.9–24.7]
mRNA MAS/GAPDH (R.U.)	0.9 [0.7–1.2]	1.2 [1.0–1.4]	1.1 [0.5–1.6]	1.2 [0.7–2.2]	1.1 [0.9–2.1]
mRNA TNF-α/GAPDH (R.U.)	14.5 [14.2–18.0]	1.2 [1.0–1.7] ^a^	1.4 [1.1–2.4] ^a^	1.3 [1.1–1.4] ^a^	1.4 [1.3–1.7] ^a^

CRF (chronic renal failure); Aox (antioxidant); Los (losartan); Aox+Los (antioxidant plus losartan); GGN1231 (antioxidant plus losartan hybrid). Data represent the median and interquartile range. ^a^ *p* < 0.05 versus CRF+vehicle.

## Data Availability

The data underlying this article will be shared upon reasonable request to the corresponding author.
